# Rehabilitation of a 51-Year-Old Patient With Miller Fisher Syndrome: A Case Report

**DOI:** 10.7759/cureus.56056

**Published:** 2024-03-12

**Authors:** Radhika Rathi, Pallavi Harjpal

**Affiliations:** 1 Department of Neuro Physiotherapy, Ravi Nair Physiotherapy College, Datta Meghe Institute of Higher Education and Research, Wardha, IND

**Keywords:** miller fisher syndrome, guillain-barré syndrome, physical recovery, neurological recovery, pain control

## Abstract

Miller Fisher syndrome is a rare and atypical variation of Guillain-Barré syndrome, which includes the clinical triad of areflexia, ataxia, and ophthalmoplegia. Miller Fisher syndrome is commonly associated with the involvement of the lower cranial and facial nerves. Miller Fisher syndrome is one of the types of Guillain-Barré syndrome. Guillain-Barré syndrome has been defined to be the foremost incapacitating form of neurological disease following the disease polio. Guillain-Barré syndrome is a broad category that encompasses several types of acute immune-mediated polyneuropathies, the most common of which is acute inflammatory demyelinating polyradiculoneuropathy. Here, we describe a case report of a 51-year-old patient who displayed the characteristic symptoms of Miller Fisher syndrome. We also describe the patient's clinical course, diagnostic method, and therapy. This case demonstrates the value of early detection, quick action in treating Miller Fisher syndrome, and the possibility of full recovery with adequate therapy. Techniques utilized in physical therapy emphasize performing everyday tasks along with strengthening muscles.

## Introduction

Neurological symptoms in Miller Fisher syndrome have been associated with anti-GQ1b IgG antibodies. Generally, it takes 10 weeks for signs and symptoms to improve completely, and almost all patients have a good prognosis. Oculomotor, dorsal ganglion neurons, and muscle spindles all express the GQ1b ganglioside [1]. Ophthalmoplegia, ataxia, and areflexia are all symptoms of the post-infectious localized variant of Guillain-Barré syndrome, which is frequently linked with face and lower cranial nerve involvement. Ataxia, ophthalmoplegia, paradoxical hyperreflexia, and alternate consciousness are all symptoms of Bickerstaff brainstem encephalitis. Fisher's syndrome has been associated with antibodies to ganglioside Q1b [2]. An upper respiratory or digestive tract disorder frequently comes before this severe polyneuropathy. In around 85-90% of all Miller Fisher syndrome patients, there is an antiganglioside antibody known as anti-GQ1b, which is self-reactive to the GQ1b ganglioside component of the nerve [3]. Globally, the most prevalent triggering infection is caused by *Campylobacter jejuni*. This associated illness with Miller Fisher syndrome, Bickerstaff brainstem encephalitis is characterized by a triad of acute bilateral ophthalmoplegia, ataxia, and encephalitis, with overlap with some forms of Guillain-Barré syndrome [4].

Acute inflammatory immune-mediated polyradiculoneuropathy, diminished or absent myotatic reflexes, distal areflexia with proximal hyporeflexia, and albumin cytologic dissociation are diagnostic criteria for Guillain-Barré syndrome. Symptoms typically include tingling, diminished strength of the muscle cells, and discomfort [5]. Often the earliest noticeable signs of Guillain-Barré syndrome are symmetrical paresthesia that grow distally. The four main subtypes of this condition are acute motor axonal neuropathy, acute motor and sensory axonal neuropathy, and acute inflammatory demyelinating polyradiculoneuropathy. Patients with a diagnosis of Guillain-Barré syndrome are treated with either plasmapheresis or IV immunoglobulin. According to reports, there are 1.2-2.3% instances of Guillain-Barré syndrome per 100,000 people yearly. Four studies conducted in Western nations indicated a winter high, whereas research from northern China, India, Bangladesh, and Latin America indicated a summer peak [6]. The prevalence of Guillain-Barré syndrome varies from 0.62 to 2.66 per 100,000 people annually, according to a recent meta-analysis, and men appear more likely than women to get Guillain-Barré syndrome [7]. The autoimmune illness of Guillain-Barré syndrome is diverse. Guillain-Barré syndrome affects 0.4 to 1.7 cases per million people annually [8]. Multiple variables that include aging, previous episodes of underlying diseases, the condition's tendency to advancement, the severity of impairment, and the amount of remedial involvement all affect the prospects for this condition. While applied around one week after the beginning of indications, plasma exchange is considered the best option and first-line therapy method for gastrointestinal bleeding syndrome. Individuals have experienced shorter times on ventilatory assistance, quicker healing, and earlier mobility due to the benefits of plasma exchange. The term multidisciplinary care adhering to Guillain-Barré syndrome implies healthcare administration involving more than two specialties such as healthcare, physiotherapy, occupational therapy, nutritional counselling, and additional healthcare providers [9]. Even though physicians might not regularly encounter individuals with Guillain-Barré syndrome daily, such individuals and the family demands seem substantial [10].

## Case presentation

Patient information

In this study, we present the case of a 51-year-old man who complained of bilateral symmetrical weakness in both upper and lower extremities over the preceding 15 days, with more of an impact on his legs than his arms. For the last six days, he had been experiencing slurred speech, tingling throughout his body, trouble eating on the right side of his mouth, and bilateral eyelid drooping. The patient had been bedridden for the previous three days due to difficulties walking, sitting, and standing, as well as a loss of mobility in his lower body. The patient had no relevant medical history of tuberculosis, bronchial asthma, diabetes, or hypertension. The assessment and inquiry confirmed that the case depicted Miller Fisher syndrome, a variant of Guillain-Barré syndrome. Consequently, the patient was admitted to Acharya Vinoba Bhave Rural Hospital, Wardha, India for specialized care in the neuro-intensive care unit before being moved to the ward. Furthermore, the patient was referred to neurophysiotherapy for further improvement in quality of life and overall functional independence.

Clinical findings

The examination process started only after obtaining the patient's verbal agreement. The head end of the patient was raised to 30 degrees while he was in a supine lying position. The patient was conscious and followed directions. His body type was ectomorph. The patient relied on his auxiliary muscles for respiration since he had trouble breathing. Both eyes showed signs of ptosis. The vitals were within normal ranges, and palpation confirmed all the examination results. Upon examination, ophthalmoplegia was determined by the paralysis of the ocular muscles. Both deep and superficial reflexes remained unaltered. In the lower limb, superficial reflexes were reduced while deep reflexes were not elicited. Clinical results showed that both lower limbs had a loss of muscle strength. Compared to the bilateral upper limb weakness, there was greater lower limb weakness.

The patient was unable to do basic everyday tasks including sitting, standing, and walking due to severe exhaustion and physical pain. In the upper limb, the superficial sensations of touch, pain, and warmth were determined to remain intact, but they were decreased in the lower limb. The tone grading scale indicated that the upper limb tone was normal, and on the lower limb, the tone was decreased (Table 1). Grade 2 power was found in both lower limbs during manual muscle testing (Table 2). The supinator jerk, triceps, and biceps all responded normally to reflex tests. There was no response on the knee and ankle (Table 3). The bilateral lower limb's hamstring and tibialis anterior muscles were found to be tight.

**Table 1:** Muscle tone (1+) represents diminished tone, (2+) represents normal tone

Muscle tone	Right	Left
Upper limb	2+	2+
Lower limb	1+	1+

**Table 2:** MMT for bilateral lower limb MMT: manual muscle testing (as per Medical Research Council grading)

Muscle	Right	Left
Hip flexors	2/5	2/5
Hip extensors	2/5	2/5
Knee flexors	2/5	2/5
Knee extensors	2/5	2/5
Ankle plantar flexors	2/5	2/5
Ankle dorsiflexors	2/5	2/5

**Table 3:** Reflexes ++: normal

Reflexes	Biceps Jerk	Triceps Jerk	Supinator Jerk	Knee Jerk	Ankle Jerk	Planter Response
Right	++	++	++	Absent	Absent	Mute
Left	++	++	++	Absent	Absent	Mute

Clinical diagnosis 

**Figure 1:**
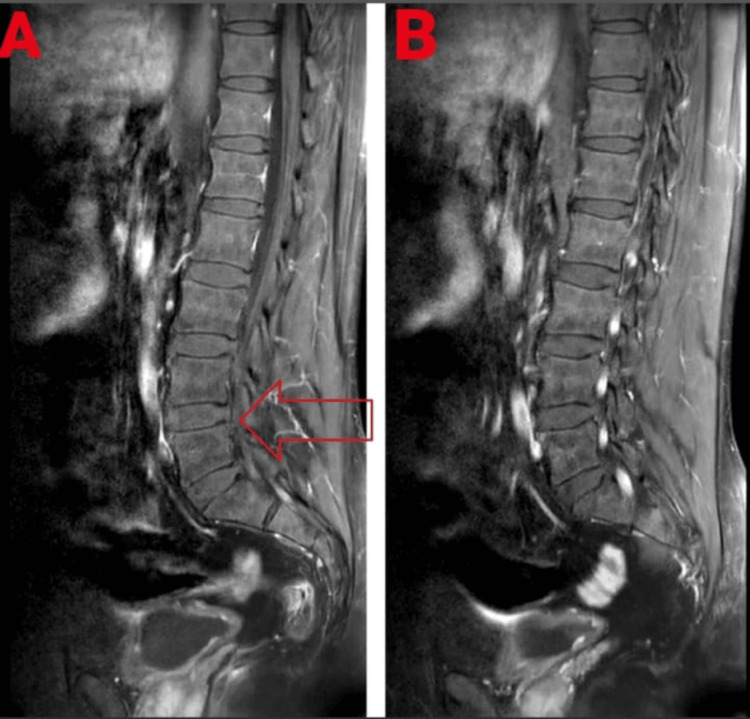
MRI scan showing (A) disc desiccation at the L4-L5 disc level, and (B) conus medularis and roots of the cauda equina depicted enhancement on contrast, raising the possibility of Guillain-Barré syndrome

**Figure 2:**
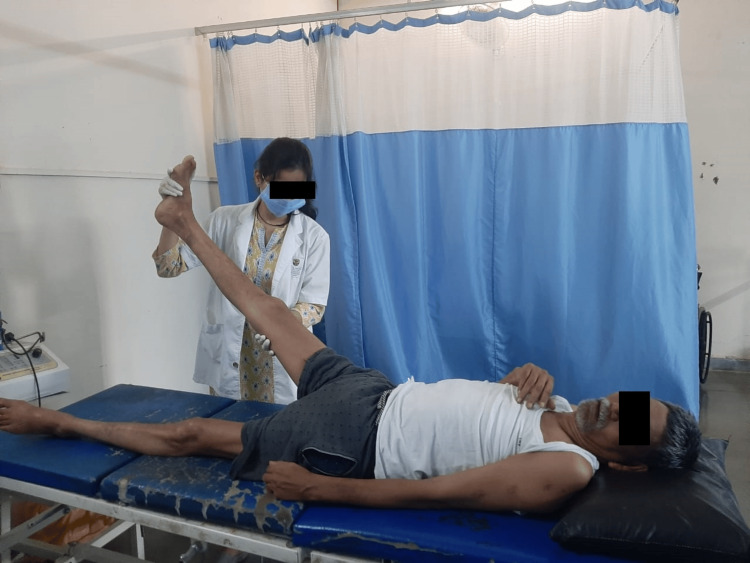
Patient performing straight leg raises

**Figure 3:**
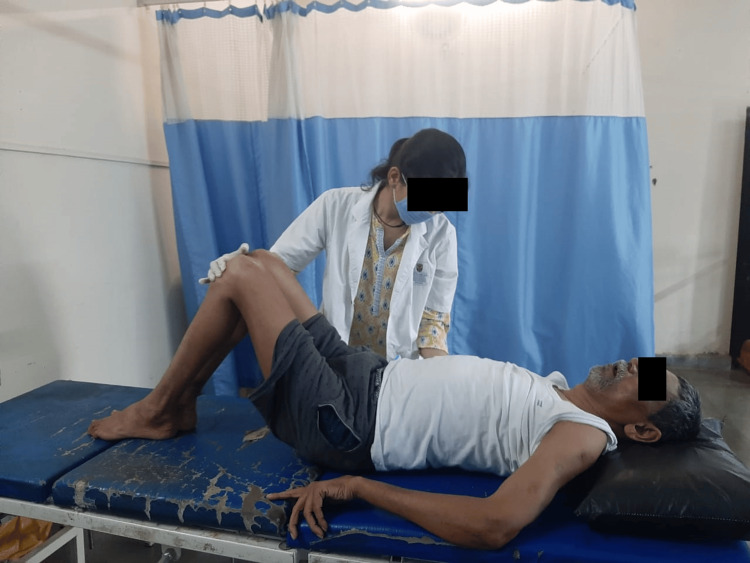
Patient performing pelvic bridging

**Figure 4:**
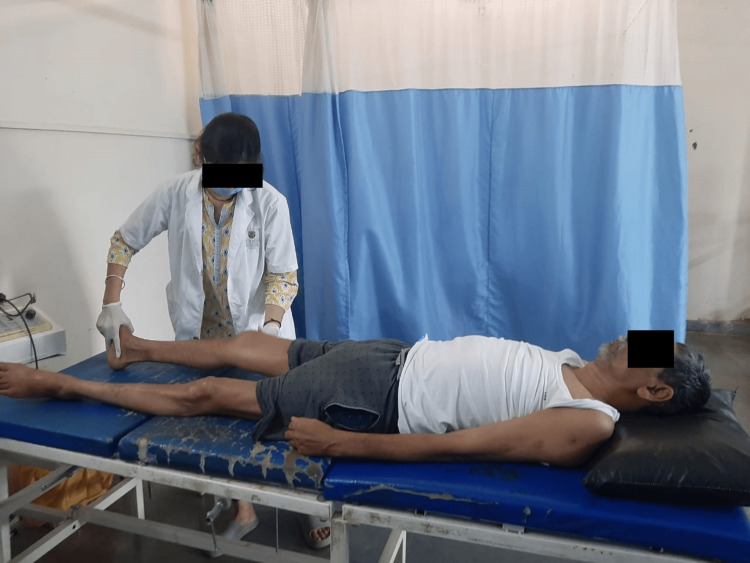
TA stretching of the patient TA: tendon Achilles

**Figure 5:**
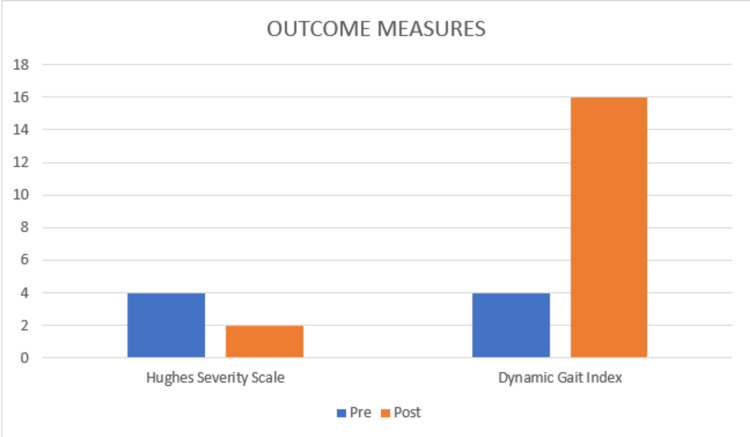
Outcome Measures - HSS and DGI HSS: Hughes severity scale; DGI: dynamic gait index

**Figure 6:**
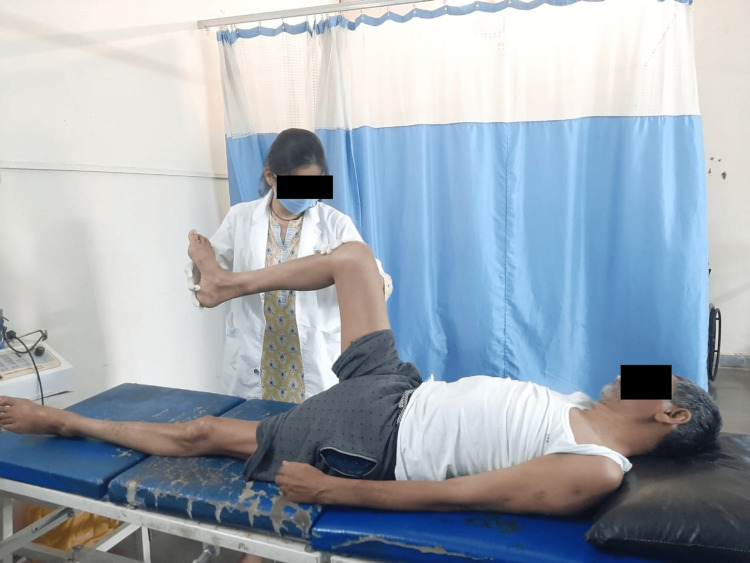
Joint approximation for the hip joint

**Figure 7:**
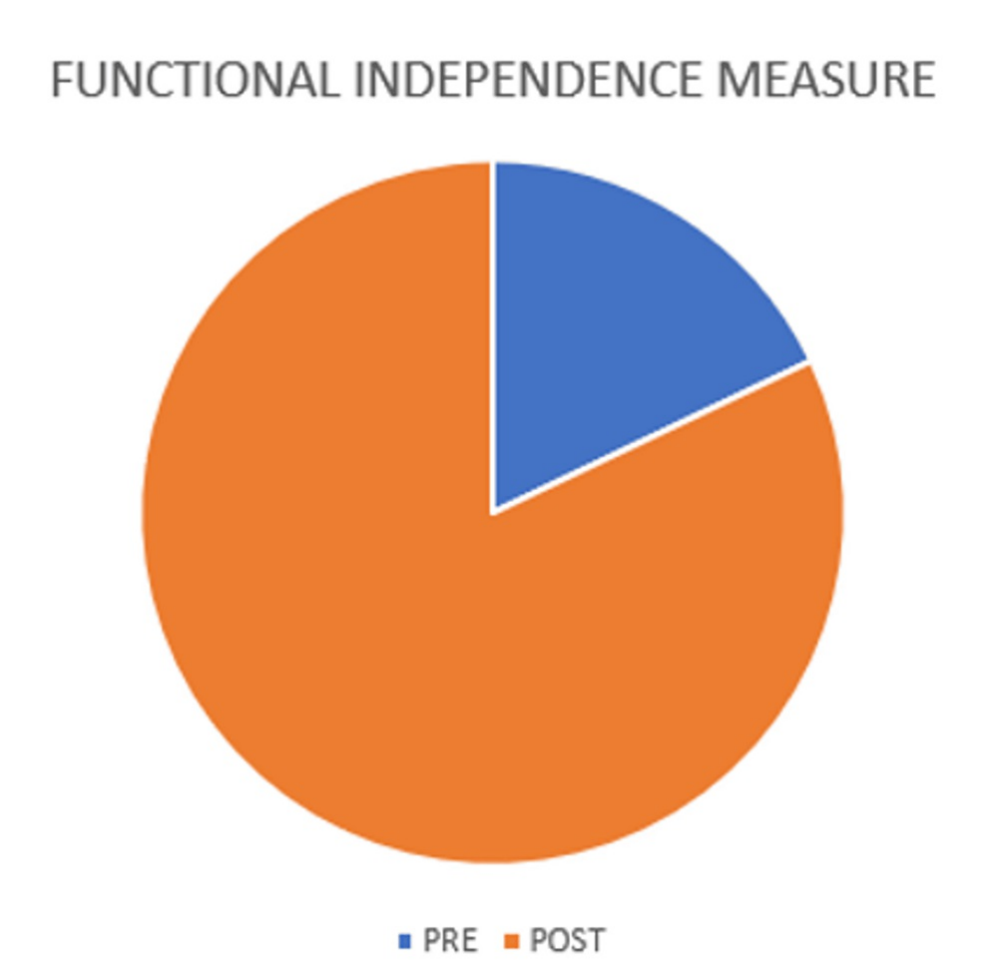
Functional independence measure

Numerous blood tests were conducted for the patient, including a peripheral smear, creatine kinase, C-reactive protein, kidney function test, and liver function test. The complete blood count was also examined. In addition, a lumbar puncture was performed to examine the cerebrospinal fluid. The ultimate diagnosis of Miller Fisher syndrome, a variant of Guillain-Barré syndrome, was made possible by the lumbar puncture and clinical findings. MRI reports showed disc desiccation at the L4-L5 disc level, and conus medullaris and roots of the cauda equina depicted enhancement on contrast, raising the possibility of Guillain-Barré syndrome. The MRI report is shown in Figure 1. Table 4 shows physiotherapy intervention. Figure 2, Figure 3, Figure 4, and Figure 5 show the exercises performed by the patient. Figure 6 and Figure 7 show the outcome measures.

**Table 4:** Physiotherapy intervention

Problem	Cause	Therapeutic Goals	Treatment
Patient and caregiver education	Muscle performance is reduced	To make the patient functionally independent	Describe the at-home workout regimen in simple terms. Describe all the red flags, as well as the dos and don'ts
Reduced air entry into the lungs	Diaphragm and intercostal muscle weakness	To improve vital capacity	Deep breathing, thoracic expansion, pursed lip breathing, diaphragmatic breathing, and glossopharyngeal breathing exercises
Preventing pressure ulcers	Prolonged immobility	To maintain proper skincare	Apply cushions for positioning. Shift every two hours. Instructions for pressure-relieving techniques (which must be performed both when sitting in a wheelchair)
Decreased range of motion	Due to weakened muscles	Stretching and active range-of-motion (ROM) exercises will engage the agonist and muscle spindle, enhancing ROM and maintaining joint integrity	After engaging in active-assisted workouts, perform passive ROM exercises, active ROM exercises for both lower limbs, and stretch
Weak back extensors and abdominals	Because of weakened abdominal muscles	To improve the strengthening of pelvic muscles	Pelvic bridging
Difficulty in breathing	Diaphragm involvement	To improve lung capacity	Incentive spirometry with inspiratory hold
Weakened lower-extremity muscles	Decreased nerve conduction and weakness as a result of the hospital stay and illness	It will enhance the power and functionality of muscles	Lower limb strength exercises with weight cuffs (starting at 1/2 kg and working up to 1 kg). Simultaneous strengthening of the hips and quadriceps
Postural deviation	Prolonged immobilization and bed rest	After two weeks, any variation in posture will come back to regular	Postural correction exercises, chest binders, positioning, and use of foam mattress
Proprioceptive impairment	Bedridden for a longer period of duration	In three weeks, proprioception will follow. On three to four weeks of gait training, the person will be able to walk with ease and at a regular cadence covered with the proper training	Joint approximation and joint compression, along with proprioceptive trainer training
Abnormal gait	Extended hospital stays, preference for proprioception	After three to four weeks of gait training, the individual can walk easily and have a normal cadence	Side leg raises, toe and heel raises, ankle dorsiflexion, single-leg stance, gait training, seated marching, squatting, and knee-to-chest movements are some of the workouts that target the quadriceps

## Discussion

As a result of its unique manifestation and uncommon evaluation, the above neurological case presents significant challenges. The surveillance of complications and crucial medical care among those diagnosed with Guillain-Barré syndrome has given way to a newly developed intensive approach (plasma exchange and intravenous immunoglobulin infusion), which reduces this condition's course and improves outcomes, particularly among individuals with severe illness. This approach for handling followed earlier research that suggested treating the root cause of the disease, treating acute neuropathy along with its consequences, and promoting the individual's sustained recovery which constitute the most important aspects of managing Guillain-Barré syndrome. Research has demonstrated to benefit critically ill patients in intensive care units, and speedy rehabilitation promotes a rapid cure. Vital signs showed extraordinary improvement, primarily attributable to the team and intensive care unit scheduled interventions [11].

Atypical Guillain-Barré syndrome is a rare version of ataxia, ophthalmoplegia, and areflexia that complies with the medical range established by Cullier. GQ1b antibodies found in the fibers of oculomotor nerves, neurons of the posterior spinal root ganglia, and neuromuscular spindles elicit an across-reaction that results in Miller Fisher syndrome following an episode of digestive problems as well as pulmonary infection or immunization [12]. Etiology of this case is likely viral (both influenza-like and gastrointestinal), which might cause a reaction of autoimmune origin that could, in a few instances, result in Guillain-Barré syndrome along with the uncommon Miller Fisher syndrome [13]. Specific subgroups of Guillain-Barré syndrome can express distinct antiganglioside indicators.

The characteristic Miller Fisher syndrome outcomes for our patient's situation had been bilateral ophthalmoplegia, hyporeflexia, and antiganglioside positive along with elevated cerebrospinal fluid. Depending on when the lumbar puncture is done, albumin-cytological separation is the usual cerebrospinal fluid finding for Guillain-Barré syndrome [14]. Common prior occurrences among patients incorporate illnesses, especially gastrointestinal and infections of the airways (83%). This clinical case study provides an example of how physiotherapy can help individuals with Miller Fisher syndrome-Guillain-Barré syndrome achieve an independent return. The severity and duration of symptoms are brought into focus, making a conventional treatment strategy more challenging to follow, and calls for additional study into how innovative therapies affect healing [15]. The functional state was evaluated using the Hughes disability scale, whereas the muscle power was evaluated using the Medical Research Council (MRC) grades [16]. Presently, there hasn't been any epidemiological research that explicitly evaluates the occurrence and rate of Miller Fisher syndrome [17]. Bersch et al. studied that based on task-focused functional training and a 16-week intensive functional electrical stimulation therapy, the outcomes show that a person with chronic Guillain-Barré syndrome with hyperreflexia can enhance their fine motor abilities [18]. It is possible to reach early independent functioning with the right medical care and therapy [19,20].

## Conclusions

In overview, the patient with Miller Fisher syndrome underwent a remarkable recovery. There were significant improvements in the individual's flexibility, interaction, and overall standard of their life. An integrated team comprising physiotherapists, occupational therapists, and speech therapists worked together for his recovery. The comprehensive approach not only restored motor function but also significantly improved the patient's overall quality of life. Physiotherapy's merits, including personalized exercise regimens and neurological retraining, proved instrumental in achieving positive outcomes. This case underscores the crucial role of physiotherapy in enhancing functional recovery and promoting optimal well-being for individuals facing neurological challenges like Miller Fisher syndrome. Although the prognosis for this patient is still being monitored, this instance highlights the significance of personalized retraining plans for uncommon neurological problems such as Miller Fisher syndrome.
